# Addressing challenges and leveraging opportunities for capacity building and the sustainable development of animal production in the south

**DOI:** 10.1093/af/vfaf027

**Published:** 2025-10-29

**Authors:** Youssef A Attia, Khalid A Asiry, Sameer A Nagadi, Asmaa F Khafaga, Rashed A Alhotan, Serena Calabrò, Bossima Ivan Koura, Manal E Shafi, Salem R Alyileili, Ahmed Galal, Samuel E Aggrey, Romdhane Rekaya

**Affiliations:** Sustainable Agriculture Production Research Group, Department of Agriculture, Faculty of Environmental Sciences, King Abdulaziz University, Jeddah 21589, Saudi Arabia; Sustainable Agriculture Production Research Group, Department of Agriculture, Faculty of Environmental Sciences, King Abdulaziz University, Jeddah 21589, Saudi Arabia; Sustainable Agriculture Production Research Group, Department of Agriculture, Faculty of Environmental Sciences, King Abdulaziz University, Jeddah 21589, Saudi Arabia; Department of Pathology, Faculty of Veterinary Medicine, Alexandria University, APIs 21944, Egypt; Department of Animal Production, College of Food and Agricultural Sciences, King Saud University, Riyadh 11451, Saudi Arabia; Department of Veterinary Medicine and Animal Production, University of Napoli Federico II, Napoli 80137, Italy; Ecole de Gestion et d’Exploitation des Systèmes d’Elevage, Université Nationale d’Agriculture, BP 43 Kétou, République du Bénin; Sustainable Agriculture Production Research Group, Department of Biological Sciences, Faculty of Sciences, King Abdulaziz University, Jeddah 21589, Saudi Arabia; Department of Laboratory Analyses, College of Food and Agriculture Sciences, United Arab Emirates University, AlAin 8221, United Arab Emirates; Poultry Production Department, Faculty of Agriculture, Ain Shams University, Cairo, Egypt; Department of Poultry Science, University of Georgia, Athens 30602-2772, GA, USA; Department of Animal and Dairy Science, University of Georgia, Athens 30602-2772, GA, USA

**Keywords:** Africa, animal production, challenges, opportunities

**Implications**

- Livestock production in South and Africa faces interconnected challenges spanning the environmental, health, economic, and social dimensions.
- South and African animal scientists can lead this transformation by tapping into genetic innovations, adopting advanced technologies, and improving feeding systems.
- Holistic and integrated approaches that consider the entire supply chain, the environmental footprint, and broader social contexts are essential.
- Prioritizing research and development in areas with significant effects, such as breeding, genetics, nutrition, and health, is crucial.
- Supportive policies and regulatory frameworks must be in place to promote sustainable practices and ensure animal welfare and food safety.
- Encouraging open collaborative partnerships between researchers, policymakers, farmers, and industry stakeholders facilitates the exchange of knowledge, resources, and workable solutions.
- Empowering women in the animal production sector should be prioritized as their roles in livestock farming are vital.

## Introduction

Animal production in the south, particularly Africa, faces several challenges that limit productivity, sustainability, and economic contribution to local and national economies. Challenges include harsh environmental conditions, climate change, limited feed resources, diseases, antimicrobial resistance, and poor animal well-being and welfare. Addressing these challenges requires concerted effort from researchers, policymakers, industry stakeholders, and farmers. Opportunities exist for the implementation of modern genetic improvement programs, adoption of advanced technologies, development of innovative and sustainable production, feeding and management systems, and enhancement of training and capacity-building approaches. Approaches that integrate research, infrastructure development, collaboration between different stakeholders, and the enhanced training and retention of local scientists within flexible and realistic policy frameworks are proposed as potential solutions. Similarly, African animal scientists are either leaving the continent, resulting in a brain drain, or facing major obstacles to resolving Africa’s livestock production challenges for several reasons, including limited funding, inadequate research and training infrastructure, and regulatory challenges. Empowering women and smallholders, increasing collaboration between different stakeholders, scaling successful models, and engaging the youth are crucial. Historically, the livestock sector in the Global South has suffered from inadequate investment and marketing innovations. Increasing investment in this sector could facilitate the adoption of innovative technologies, boost research funding, empower women and youth, and promote infrastructure development, thus leading to more efficient value chains. However, no major progress will be made without a well-trained and engaged animal scientist community working in synergy with different stakeholders. Addressing these challenges is key to progress and innovation, fighting poverty, job creation, and creating a circular economy.

## Contemporary Issues in Animal Science Across the South

**Figure 1.**
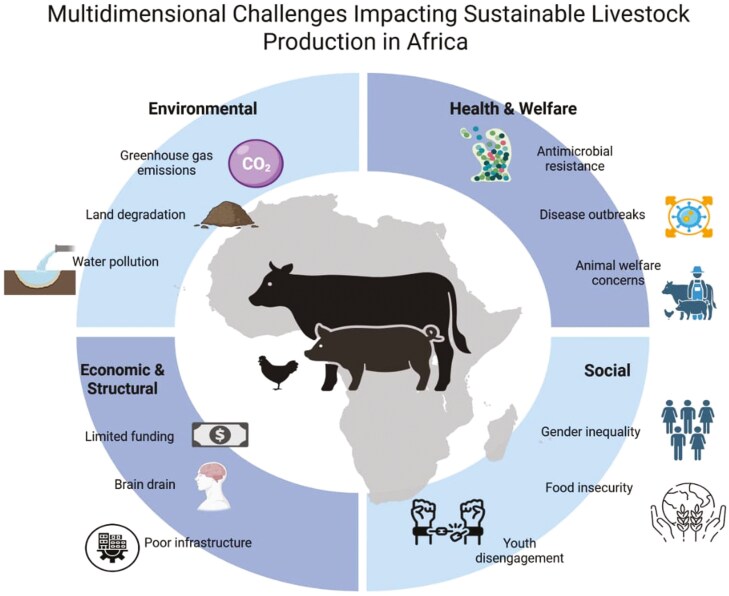
Multidimensional challenges impacting sustainable livestock production in South.

Livestock production in Africa faces interconnected challenges spanning the environmental, health, economic, and social dimensions (Figure 1).

Addressing these issues requires holistic strategies to mitigate risk and enhance sustainability. One of the greatest concerns is the environmental impacts of livestock production. Livestock is a major contributor to greenhouse gas emissions, land degradation, and water pollution, making the adoption of more sustainable practices both important and urgent. The coronavirus disease (COVID-19) pandemic has affected the environment, animal health, and food security. Reduced human mobility improves air and water quality, which has a positive impact on the quality of life of honeybees (Attia et al., 2024). However, COVID-19 has also drastically impacted livestock production, affecting countries’ ability to prevent and control animal diseases and increasing global poverty, thus becoming a threat to the sustainability of global food security (Alshubaith et al., 2022). Rising temperatures and shifting rainfall patterns have affected the health, productivity, and geographic distribution of livestock. Livestock is one of the drivers of climate change (Gninkplékpo et al., 2024). Changes in land use and reduced water sources have led to increased competition between pastoralists and farmers, prompting the call for adaptive strategies that can help build resilience across the sector (Cheng et al., 2022). Thus, antimicrobial resistance is a pressing issue. The excessive and inappropriate use of antimicrobials in animal farming not only affects animals but also represents a real threat to human health. Thus, it is crucial to promote the use of antibiotics and explore alternative approaches to antibiotic use (Hafez et al., 2021).

Additionally, there is a clear need for continuous education and training for those working directly with animals. Without proper knowledge and updated practices, even the best strategies can fall short (Hafez and Attia, 2020). Finally, many farms have struggled with inadequate hygiene and control measures. Addressing this issue through more precise and enforceable farm management strategies is key to reducing disease risk and improving overall productivity (OIE, 2020b; Attia et al., 2022a; Attia et al., 2022b).

## Multidisciplinary Opportunities for Animal Science in South and Africa

**Figure 2.**
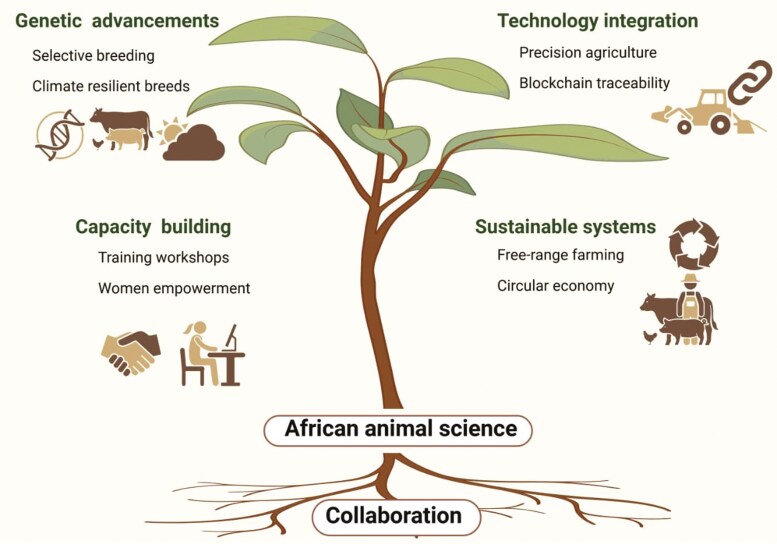
Multidisciplinary opportunities for Animal Science in South.

Despite its many challenges, the farm animal industry in Africa has tremendous potential for growth and innovation. With appropriate strategies, the sector can move towards more sustainable and productive livestock systems. African animal scientists are already in a strong position to lead this transformation by tapping into genetic innovation, adopting advanced technologies, improving feeding systems, and encouraging cross-sector collaboration (Figure 2).

Empowering women and youth along with thoughtful policy reforms will further amplify the impact of these efforts. Genetic improvement is a promising area in which techniques such as selective breeding and the use of genomics information can significantly boost productivity, enhance disease resistance, and improve adaptability to climate change (Dawkins, 2012; Nicol et al., 2021; Djikeng et al., 2025). Simultaneously, cutting-edge technologies—from precision agriculture to biotechnology, digital tools, and artificial intelligence– can help increase efficiency, reduce waste, and make production systems more sustainable (Hashem et al., 2021; Vlaicu et al., 2024). In addition, organic and free-range farming are gaining traction and have the potential to meet the growing demand of niche markets while aligning with evolving consumer preferences (Hafez et al., 2021; Hashem et al., 2021; Rahman et al., 2022).

Feeding strategies also play a key role, especially in regions where climate conditions make consistent and high-quality feed availability challenging. One eco-friendly solution is the use of agro-industrial residues in animal diets, which not only reduces the environmental impact but also boosts productivity in a sustainable manner (Kiatti et al., 2024). The use of crop residues is a major feeding strategy in sub-Saharan Africa, contributing to nutrient recycling in agro-ecosystems (Koura et al., 2024).

To support these advancements, an improved long-term investment in capacity building is essential. Strengthening education and training programs will enhance the skills and knowledge of everyone involved in the sector, from scientists and students to farmers and technical staff (Hafez and Attia, 2020; Hashem et al., 2021). The adoption of continuous education, updated curricula, and expanded hands-on training will likely aid the innovation resilience of the animal production sector in Africa. This can be achieved by connecting research in animal sciences with national livestock development strategies, and the private sector needs to address key problems at the national and regional levels (Vilas-Boias et al., 2022).

## Innovative Solutions for Animal Science in South

To address these challenges and capitalize on opportunities, we suggest the following solutions:

1. Adoption of holistic and integrated approaches that look beyond isolated issues and consider the entire supply chain, environmental footprint, and broader social context of animal production (Hafez et al., 2021; Hashem et al., 2021; Rahman et al., 2022). The interconnectedness of different elements of the livestock production system offers a more effective decision-making and problem-solving approach, as suggested by Cummings et al. (2023).
2. Prioritizing research and development in areas with large impacts, such as breeding, genetics, nutrition, and health, is essential to drive meaningful improvements and spark innovation across the sector (Dawkins et al., 2012; Hafez and Attia, 2020; Shehata et al., 2022; Djikeng et al., 2025). This includes the development of strategies that promote indigenous breeds and the sustainable utilization of innovative feed solutions, such as insect-based proteins, agro-industrial by-products, and algae-based feeds (Camara et al., 2019; Ouédraogo et al., 2021; Van der Poel et al., 2020). However, innovation alone has been insufficient.
3. Supportive policies and regulatory frameworks must be in place to promote sustainable practices and ensure animal welfare and food safety. These frameworks create a foundation for sustainable improvement and progress (Hashem et al., 2021; Nicol et al., 2021; Vlaicu et al., 2024; Djikeng et al., 2025).
4. Encouraging open collaborative partnerships between researchers, policymakers, farmers, and industry stakeholders facilitates the exchange of knowledge, resources, and workable solutions (Hafez and Attia, 2020). In addition, promoting South-South collaboration will enable exchanges of contextual experiences and learning from studies, experiments, and success stories in countries in the Global South (Mawdsley, 2019).
5. Agricultural education should be included in pre-high school curriculum to ensure future animal production farmers are abreast with animal-related issues, husbandry practices, and limitations.
6. Continuous training and updated curricula are needed to equip the next generation of animal scientists, trainers of the trainers, trainers of the farmers, and farmers with the tools and skills to navigate a rapidly changing agricultural landscape (Wong, 2023). Simultaneously, proven approaches must be scaled up quickly and effectively. Without scaling, even the best local solutions will have a limited effect (Sharaf et al., 2024 ; 2025).
7. Empowering women in the animal production sector should be a priority. Women’s roles in livestock farming are vital, and investing in their access to training, resource availability, and decision-making opportunities will significantly strengthen this system (Wong, 2023. Youth also need to be empowered, especially those holding degrees in animal science, through job opportunities and continuous support and training (Nchanji et al., 2023). This will lead to a reduction in brain drain and an increase in productivity.

## The Status of Animal Scientists in South and Africa

Animal scientists in South Africa navigate a complex landscape filled with challenges that directly affect their ability to conduct research, develop innovative technologies, and enhance animal productivity and welfare. One of the most pressing issues is lack of funding. Research in animal science across many African countries tends to be grossly underfunded, which naturally limits the scale and effectiveness of scientific projects (OIE, 2020a). In addition to financial constraints, there is also an issue of limited infrastructure. Many institutions lack access to up-to-date facilities or modern equipment, making it difficult to conduct high-quality, cutting-edge, and long-term research (FAO, 2006; Wong et al., 2023). Another major concern is brain drain. Talented animal scientists often leave the continent to search for better professional opportunities. This migration of expertise results in a significant loss of skilled professionals, who could otherwise contribute to the local development of the field (IOM, 2019; Hafez and Attia, 2020). International collaboration, while becoming increasingly important, can be difficult to establish and maintain. Scientists may face barriers, including limited access to global research networks, language barriers, and cultural challenges, which make it difficult to sustain (Hafez and Attia, 2020; Wong et al., 2023). Finally, navigating the regulatory environment is a major challenge on its own right. Based on strict laws on animal welfare, environmental protection, and complex food safety standards, regulatory hurdles can slow progress and make research and implementation more complicated (FAO, 2006; SADC, 2019; SASAS, 2021; Wong, 2023).

## Strategic opportunities for Animal Scientists in South and Africa

Despite many obstacles, animal scientists in Africa have been tapping into exciting opportunities for growth and development. Genetic improvement is one of the most promising research areas. Through selective breeding and genomics, it is possible to enhance productivity, increase resistance to disease, and assist animals in better adapting to the effects of climate change (Oloo et al., 2023; Djikeng et al., 2025). Genomics offers the opportunity to overcome some of the limitations of data collection (e.g., pedigree information, reduce the generation interval, and increase breeding value estimation, resulting in faster genetic progress.

Technology has played a major role in this transformation. The adoption of precision agriculture tools, biotechnology, and digital innovations can improve efficiency, reduce waste, and make farming practices more sustainable (Vlaicu et al., 2024). Furthermore, it allows for an automated or quasi-automated collection of novel and hard-to-measure phenotypes and endophenotypes, opening possibilities for new selection goals. Alongside these advancements, there is a growing push toward more innovative production systems (e.g., organic, free-range, and backyard production) that are increasingly appealing to consumers looking for ethical and environmentally friendly options (Hollifield et al., 2024).

Investment in education and training not only equips scientists and farmers with the skills they need but also drives innovation across the industry (Wong, 2023). Empowering women is key to transformative changes. When women are given access to financial resources, training, and leadership opportunities, they bring immense value to the livestock sector, improving both animal husbandry practices and overall sustainability (Wong, 2023). A collaborative, multisectoral approach is essential for advancing sustainable livestock systems. This means bringing together government agencies, research institutions, producer organizations, development agencies, civil society, and the private sector. By working together, these groups can tackle complex issues ranging from health management to entrepreneurship (Wong, 2023;  AU-IBAR, 2019). New tools, such as genomic selective breeding programs and

Information technology (IT-based solutions [e.g., mobile apps are also gaining ground]). These innovations make it easier to manage livestock and bee farming and, when scaled effectively alongside training initiatives, can have a huge impact (Dawkins et al., 2012; Djikeng et al., 2025). However, to fully harness these solutions, both public and private investments are needed, supported by smart policies and cross-sector collaboration (Wong, 2023).

Climate change resilience is another critical research area. Therefore, finding innovative ways to reduce the carbon footprint of livestock systems is becoming increasingly urgent (Attia et al., 2024; IPCC, 2022). For ruminant production, there is a need to define sustainable strategies to optimize feeding practices. This includes improving data on the nutritional value of local feed resources in the south. Farmers’ traditional knowledge of forage species, especially those that thrive during drought, can aid in the identification of climate-resilient options. Understanding and cultivating these species, particularly in coastal and climate-stressed areas, may play a major role in sustaining dairy production (Koura et al., 2022). Promoting climate-smart technologies and practices is not only good for the planet but also increases productivity and builds stronger and more adaptable systems (Kabato et al., 2025; Wong, 2023).

Promote indigenous breeds that are more resilient and can meet the current changing environment and production in harsh environments (Mapiye et al., 2020; Aggrey et al., 2020; Rekaya and Aggrey, 2020; Aggrey and Rekaya, 2022). Of course, no progress has been made without the support of policymakers. Their decisions shape the landscape for livestock development; therefore, it is vital that they understand the value of sustainable practices and create a policy environment that encourages private investment (Wong, 2023).

Finally, the long-term sustainability of the livestock sector depends on the younger generations. Encouraging youth to participate in agribusiness through mentorship, training, and entrepreneurial support is essential for keeping the sector innovative, dynamic, and future-ready (Wong, 2023).

## Possible Solutions for Animal Scientists in South and Africa

To address these challenges and capitalize on opportunities, we consider the following solutions:

1. Increased funding: Governments, international organizations, and private donors can provide more funding for animal science research in South Africa, which can help improve the research infrastructure and supportive logistic networks.
2. Capacity-building Animal Scientists in South Africa can participate in training and capacity-building programs to improve their skills and knowledge in areas such as genomics, precision agriculture, and artificial intelligence.
3. International collaboration: Animal scientists in South Africa can collaborate with international partners to access global research networks, share knowledge, and leverage resources.
4. South-South collaboration: Animal scientists can network and share knowledge and facilities in the southern countries.
5. Policy and regulatory frameworks: Governments can establish supportive policies and regulatory frameworks to promote sustainable practices, animal welfare, and food safety.

## Conclusion

The livestock industry in South Africa is instrumental in transforming the continent’s food system and offers a range of benefits beyond food production. This sector is vital for boosting food security, enhancing nutrition and investment, creating new jobs and income opportunities, supporting rural communities’ livelihoods, and reducing poverty. Achieving sustainable livestock systems requires a comprehensive approach that integrates the various key elements. Collaboration among scientists and stakeholders such as farmers, researchers, policymakers, and industry participants is essential for tackling the complex challenges faced by the sector. Scaling up successful interventions and embracing climate-smart innovations are crucial for boosting productivity, while reducing the environmental impact of livestock and poultry. Greater investment in research, infrastructure, and capacity building is required to drive innovation and to enhance sectoral efficiency. Developing inclusive policies that address the needs of smallholder farmers and marginalized communities is crucial for ensuring equitable growth in the livestock sector. Empowering women and youth through targeted initiatives and training programs can unlock untapped potential and stimulate innovation within the industry. By concentrating on these critical areas, Africa can establish resilient local livestock systems that not only contribute to food security but also mitigate environmental impacts and promote economic development. However, the animal science and science sectors in Africa have encountered numerous challenges, including limited access to resources, inadequate infrastructure, and the effects of climate change. Overcoming these hurdles requires concerted efforts from all stakeholders to utilize research, technology, and policy interventions to create sustainable solutions. By seizing emerging opportunities and implementing effective strategies, the livestock sector can become a driving force in Africa’s journey towards economic prosperity, improved food security, and environmental sustainability.

## Author Biography

****

**Professor Youssef A. Attia** is a Distinguished Professor of Poultry Nutrition and the chairman of the Sustainable Agriculture Production Research Group at King Abdulaziz University, Jeddah, Saudi Arabia. Prof. Attia gained a Doctor of Philosophy in Agricultural Science (Poultry Production) from the University of Zagazig, Egypt, University of Georgia, Athens, Georgia, USA (April 1993). Most of the PhD work was done through the channel system at the University of Georgia, Poultry Science Department, Athens, Georgia, USA, from June 1990 to December 1992. Professor Attia published 350 articles, including 16 books, five book chapters, five university books, and one book translated to Arabic. In addition, he has been recognized as a reviewer in approximately 90 ISI journals and a funding body for local and international scientific organizations. He is a capacity-building professor who has trained many graduate and postgraduate students and postdocs throughout the world, and maintains collaborative partnerships in Africa, Asia, Europe, and the United States. Professor Attia attended approximately 40 national and international conferences, congresses, and workshops. He is also a member of many editorial boards, peer-reviewed journals, the Scientific and Organized Committee of many conferences, and the Congress. Prof. Attia gained many scientific awards and was recognized by the Khalifah Award 12 cycle (2018–2019) as being a distinguished Professor in the Arab World. Prof Attia gained Rashid bin Humaid Award, sessions 38 and 40 for Culture and Science. The second place is on the general level of the Environmental Public Speech Competition for the Emirates Environment Group in Dubai, November 20–24, 2023. He ranked as #1st King Abdulaziz University, #1 in Saudi Arabia, #20 in Asia, and # 205 in the world, according to the World AD Scientific Index 2025. He Lasted in the World’s Top 2% Scientists List of Stanford University 2020-2024. He is among the top 1% of the Plant and Animal Science and Agriculture and Biological Science reviewers. He horned an editorial board member of many international Journals, AJAS 2020 Best Reviewers Award, the best reviewer for the Animal Nutrition journal, a section editor of the Italian Journal of Animal Science, and a guest editor of many special issues. Member of the Review Committee of Abdul Hameed Shoman Arab Researcher Award 2015.

**Professor Khalid A. Asiry** is an agricultural scientist. He is a Full Professor at Agriculture Department, King Abdulaziz University in Saudi Arabia, where he teaches a variety of agricultural science courses. His current research focuses on managing and producing livestock, with the goals of increasing feed efficiency, facilitating climate change adaptation, and improving livestock productivity. Prof. Khalid has many publications in peer-reviewed journals, and he reviews many international peer-reviewed journals. He also oversees all academic operations as head of the Agriculture Department, including the creation of new programs, the Agriculture Research Center, and different research activities.

**Dr. Sameer A. Nagadi** is an Associate Professor of Animal Nutrition in the Agriculture Department, and a member of the Sustainable Agriculture Production Research Group, Faculty of Environmental Sciences, King Abdulaziz University, P.O. Box 8020, Jeddah, Saudi Arabia.

**Professor Asmaa Khafaga** is a professor at Alexandria University, Egypt. She received the State Encouragement Prize in Agricultural Sciences and Alexandria University Award for Scientific Encouragement in 2020. According to the Stanford University rankings, she has been listed among the world’s top 2% scientists from 2020 to 2024. Prof. Khafaga has authored over 170 published papers and 17 book chapters. Her research focuses on pathology, especially disease mapping, cancer, neurodegenerative diseases, and the use of innovative alternative and complementary medicines to alleviate animal toxicosis. She has served as a member of the Animal and Fisheries Research Council (2020–2024) and is currently a member of the Research Ethics Council at the Academy of Scientific Research and Technology, Egypt, as well as the local advisory council for developing Arab journals—a collaboration between Elsevier, the League of Arab States, and the Union of Arab Universities.

**Dr. Rashed A. Alhotan** is an Associate Professor in the Department of Animal Production, College of Food and Agricultural Sciences, King Saud University. He has published more than 100 articles and has reviewed articles in many peer-reviewed journals. His research focuses on feed formulation, amino acid requirements, and heat-stress mitigation in poultry.

**Professor Serena Calabrò** is a Doctor in Veterinary Medicine, PhD in Animal Breeding Science, and Full Professor in Animal Nutrition at the Department of Veterinary Medicine and Animal Production (DMVPA), University of Napoli Federico II (Italy). The main research activity involves the use of an in vitro gas production technique to study fermentation characteristics and estimate the nutritive value of ruminant and non-ruminant feedstuffs, with reference to the estimation of environmental impact (in vitro measurement of methane production). Authors of 112 indexed publications (h-index: 27). She participated in research programs of international, national, and local interests. She has lectures on Animal Nutrition for undergraduate and post-graduate students. She has been a supervisor for the degree thesis in Animal Nutrition of several students in Animal Production and a tutor of four PhD students in Veterinary Science. She is a coordinator of a 3-year bachelor’s degree course in Animal Production, a component of the School of Agronomy and Veterinary Medicine, and of the College of Doctoral School in Veterinary Science. She is the promoter of the Erasmus student exchange program at the University of Agricultural Sciences and Veterinary Medicine, Bucharest. She is part of the Unina Committee of MUNA (Mediterranean and Middle East University Network Agreement) and the coordinator of the agreement for cooperation between DMVPA and the National University of Agriculture (Republic of Benin). She is a Co-editor of the Science Magazine Un Mondo di Bufale.

**Dr. Bossima Ivan Koura** is an animal scientist and Associate Professor in Animal Nutrition and Farm System at the National University of Agriculture (UNA) in Benin, who teaches various courses related to animal husbandry. Ivan conducted research on livestock production and management, aiming to enhance livestock productivity, improve feed efficiency, and facilitate adaptation to climate change. His current research focuses on (i) climate-smart livestock systems, including the development of more effective feeding strategies and increasing the production of indigenous cattle and poultry breeds, (ii) sustainable utilization of grassland and forage resources involving IoT and sensor tools, and (iii) nutritional interventions (such as innovative feeds and natural feed additives) to reduce methane emissions and improve animal product quality. Additionally, Ivan serves as the academic coordinator of the TVET Education Program (UNA-Benin and UKZN-South Africa). He is also the deputy coordinator of the PhD program in animal and fisheries science at the UNA.

**Dr. Manal E. Shafi** is an Associate Professor at Department of Biological Sciences, Zoology, King Abdulaziz University, Jeddah, Saudi Arabia, and a member of the Sustainable Agriculture Production Research Group. E-mail: Meshafi@kau.edu.sa.

**Dr. Salem R. Alyileili** is the Head of the Main Analytical Laboratory in Abu Dhabi, The United Arab Emirates. He gained a Master’s in Environmental Sciences -Aridland Department –College of Food and Agriculture—United Arab University, 2011, and in Animal Sciences and PhD in Animal Sciences -Aridland Department –College of Food and Agriculture—United Arab University, 2018. He has published 15 papers in peer-reviewed, high-ranked journals. He had three Patents: Date Pit Composition for Treatment of Animals, the European Union Patent and Trademark Office, Publication No. EP2586318 (A1). The EU Patent Office granted patents on April 16, 2014. “Date Pit Composition for Treatment of Animals.” The United States Patent and Trademark Office. The US Patent Publication No. US 8,968,729 B2. The US Patent Office granted a patent on March 3, 2015. “Date Pit Composition for Treatment of Animals.” The Hong Kong Patent and Trademark Office Patent Publication No. HK patent No. HK1184642 B. The Hong Kong Patent Office granted patents on January 9, 2015.

**Professor Ahmed Galal** is a Full Professor of Poultry breeding and former Dean of the Faculty of Agriculture, Ain Shams University, and current Dean of the Higher Institute of Agricultural Cooperation, Shubra, Egypt.

**Professor Samuel E. Aggrey** is a Distinguished Research Professor in Genetics and Genomics at the University of Georgia, USA. He is also a Richard B. Russell Endowed Chair and Professor at the Department of Poultry Science, University of Georgia, Athens, GA, USA. Professor Aggrey obtained his BSc degree in Animal Science at the University of Ghana, Legon, and MSc with Distinction at Wageningen University in the Netherlands, and PhD in Quantitative Genetics and Poultry Breeding at the University of British Columbia, Vancouver, Canada. He is also an affiliate scientist at Biosciences East and Central Africa (International Livestock Research Institute, Kenya). He is the Capacity Building Pillar Chair for the African Animal Breeding Network. Among the many interests of Professor Aggrey is to breed adaptive chickens in harsh environments to improve nutritional security and reduce poverty. He serves on the High-Level Think-Tank to Respond to Technological Issues in African Agriculture, and the African Union’s Working Group on Animal Seeds of the African Seed and Biotechnology Partnership Platform. Professor. Aggrey is a co-editor of “Poultry Genetics, Breeding and Biotechnology” and the lead editor for “Advances in Poultry Genetics and Genomics”. He has authored several book chapters and over 200 peer-reviewed publications. He has trained several postgraduate students and postdocs throughout the world and has maintained collaborative partnerships in Africa, Asia, and Europe. His notable awards include the Jaap Memorial Lectureship at the Ohio State University, the D.W. Brooks Award for Excellence in Research, Career Development Recognition (the University of Georgia), The Broiler Research Award (Poultry Science Association), Changxin Chu Fund (China Agricultural University Educational Foundation/World Poultry Science Association), Carnegie Fellowship (Carnegie Institute), and World’s Poultry Science Association P. B. Siegel Research Award, D. W. Brooks Award for Excellence in International Agriculture, and also for Excellence in Diversity.

**Professor Romdhane Rekaya** is a Professor of Animal Breeding and Genetics at the University of Georgia. He received an agriculture engineer degree from the High Institute of Agriculture, Tunisia, a Master of Science from the International Center for Advanced Studies in Mediterranean Agriculture of Zaragoza, Spain, and a PhD from the Polytechnic University of Madrid, Spain. Dr. Rekaya has extensive experience in quantitative genetics, genomics, biostatistics, and bioinformatics. His research program is centered on the development of statistical and computational tools for analysis of big genetic and genomic data sets with application in livestock, poultry, and human. Dr. Rekaya’s research has been supported by several federal, state, and private funding agencies, including the USDA NRI, AFRI, and NIFA programs, NAAB, the Georgia Agricultural Commodity Commission for beef, Cobbs-Vantress, Inc., Merial, Inc., and Tyson Foods. He has published 134 refereed journal articles, 4 book chapters, and over 170 abstracts and proceedings. Dr. Rekaya is an adjunct professor at the Statistics Department and an associate faculty member at the Institute of Bioinformatics at UGA. He taught several international courses in Canada, Norway, Brazil, and Kenya.

## References

[CIT0002] Aggrey, S., and R. Rekaya. 2022. Poultry breeding. In: Meyers, R.A. (eds) Encyclopedia of sustainability science and technology, New York, NY: Springer; p. 181–190. doi: https://doi.org/10.1007/978-1-4939-2493-6_1118-1

[CIT0001] Aggrey, S.E., P.B. Siegel, and R. Rekaya. 2020. Poultry breeding for sustainability and plasticity in functional traits: reality or fiction in the midst of conflicting interests. In: Aggrey, S.E., H. Zhou, M. Tixier-Boichard, and D. Rhoads, editors. Advances in poultry genetics and genomics. Sawston, England: Burleigh Dodds Science Publishing. ISBN 9781786763242.

[CIT0003] Alshubaith, I.H., S. Alhajri, A. Alhajri, R.A. Alsultan, E.I. Azhar, L.S. Al Solami, B. El-Hussieny, M.C. de Oliveira, and Y.A. Attia. 2022. The impact of COVID-19 on the sustainability of the environment, animal health and food security, and safety. Environ. Sci. Pollut. Res. 29:70822–70831. doi: https://doi.org/10.1007/s11356-022-22468-0 PMC9446590 36066798

[CIT0006] Attia, Y.A., K. Aldhalmi, I.M. Youssef, F. Bovera, V. Tufarelli, M.E. Abd El-Hack, K.H. El-Kholy, and M. Shukry. 2024. Climate change and its effects on poultry industry and sustainability. Discov. Sustain. 5(3):397. doi: https://doi.org/10.1007/s43621-024-00627-2

[CIT0004] Attia, Y.A., G.M. Giorgio, N.F. Addeo, K.A. Asiry, G. Piccolo, A. Nizza, C. Di Meo, and F. Bovera. 2022a. COVID-19 pandemic: impacts on bees, beekeeping, and potential role of bee products as antiviral agents and immune enhancers. Environ. Sci. Pollut. Res. 29:9592–9605. doi: https://doi.org/10.1007/s11356-021-17643-8 PMC8736297 34993785

[CIT0005] Attia, Y.A., M.T. Rahman, M.J. Hossain, S. Basiouni, A.F. Khafaga, A.A. Shehata, and M.H. Hafez. 2022b. Poultry production and sustainability in developing countries under the COVID-19 crisis: lessons learned. Animals 12(5):644. doi: https://doi.org/10.3390/ani12050644 35268213 PMC8909689

[CIT0007] AU-IBAR. 2019. Animal health strategy for Africa (AHSA) 2019–2035: a framework for delivering a sustainable animal health system that meets global standards. http://repository.au-ibar.org/handle/123456789/539

[CIT0008] Camara, Y., M. Ciss, N. Moula, M.M. Sissokho, F. Farnir, and N. Antoine-Moussiaux. 2019. Determinants of breeders’ participation in an indigenous cattle breeding program. Agron. Sustainable Dev. 39(5):1–12. doi: https://doi.org/10.1007/s13593-019-0591-1

[CIT0009] Cheng, M., B. McCarl, and C. Fei. 2022. Climate change and livestock production: a literature review. Atmosphere 13(1):140. doi: https://doi.org/10.3390/atmos13010140

[CIT0010] Cummings, D.B., J.T. Groves, and B.L. Turner. 2023. Assessing the role of systems thinking for stocker cattle operations. Vet. Sci. 10(2):69. doi: https://doi.org/10.3390/vetsci10020069 36851373 PMC9961819

[CIT0011] Dawkins, M.S., and R. Layton. 2012. Breeding for better welfare: genetic goals for broiler chickens and their parents. Anim. Welf. 21(2):147–155. doi: https://doi.org/10.7120/09627286.21.2.147

[CIT0012] Djikeng, A., V.E. Olori, I. Houaga, S.E. Aggrey, O. Mwai, E.M. Ibeagha-Awemu, R. Mrode, M.G.G. Chagunda, C.K. Tiambo, R. Rekaya, et al 2025. The African animal breeding network as a pathway towards genetic improvement of livestock. Nat. Genet. 57(3):498–504. doi: https://doi.org/10.1038/s41588-025-02079-4 39930083

[CIT0013] FAO. 2006. Livestock’s long shadow– [accessed April 24, 2025]. https://www.fao.org/4/a0701e/a0701e00.htm

[CIT0014] Gninkplékpo, E.L.R., B.I. Koura, P. Lesse, I. Toko, D. Demblon, M.R. Houinato, and J.F. Cabaraux. 2024. Small ruminant farmers’ feeding strategies to cope with climate change across five agroecological zones of Benin, West Africa. Heliyon 10(21):e37737. doi: https://doi.org/10.1016/j.heliyon.2024.e37737 39553670 PMC11566677

[CIT0015] Hafez, H.M., and Y.A. Attia. 2020. Challenges to the poultry industry: current perspectives and strategic future after the COVID-19 outbreak. Front. Vet. Sci. 7:516. doi: https://doi.org/10.3389/fvets.2020.00516 33005639 PMC7479178

[CIT0016] Hafez, H.M., Y.A. Attia, F. Bovera, M.E. Abd El-Hack, A.F. Khafaga, and M.C. de Oliveira. 2021. Influence of COVID-19 on poultry production and environment. Environ. Sci. Pollut. Res. 28(33):44833–44844. doi: https://doi.org/10.1007/s11356-021-15052-5 PMC8269985 34244934

[CIT0018] Hashem, N.M., E.M. Hassanein, J.F. Hocquette, A. Gonzalez-Bulnes, F.A. Ahmed, Y.A. Attia, and K.A. Asiry. 2021. Agro-livestock farming system sustainability during the COVID-19 era: a cross-sectional study on the role of information and communication technologies. Sustainability 13(12):6521. doi: https://doi.org/10.3390/su13126521

[CIT0042] Hollifield, M.K., C.-Y. Chen, E. Psota, J. Holl, and D. Lourenco. 2024. Estimating genetic parameters of digital behavior traits and their relationship with production traits in purebred pigs. Genet. Sel. Evol. 56(1):29. doi: https://doi.org/10.1186/s12711-024-00902-w 38627636 PMC11022375

[CIT0019] IOM. 2019. Migration and development in Africa– [accessed April 24, 2025]. https://publications.iom.int/system/files/pdf/pub2023-132-r-iom-au-africa-migration-report-second-edition_0.pdf

[CIT0020] IPCC. 2022. Climate change 2022: impacts, adaptation and vulnerability– [accessed April 28, 2025]. https://noc.ac.uk/news/noc-contributes-latest-ipcc-report-assessing-impacts-climate-change?gad_source=1andgbraid=0AAAAApOYD3yUDS7SZ40TK28gZrpSTkMInandgclid=CjwKCAjw--.K_BhB5EiwAuwYoyowQ5gGnpTfG0OmWoHsDgPLw3KxFKzq8p6vNSlJwAHWphotSnO5w3hoCLjIQAvD_BwE

[CIT0043] Kabato, W., G.T. Getnet, T. Sinore, and A. Nemeth. 2025. Towards Climate-Smart Agriculture: Strategies for Sustainable Agricultural Production, Food Security, and Greenhouse Gas Reduction. Agronomy 15(3):565. doi: https://doi.org/10.3390/agronomy15030565

[CIT0021] Kiatti, D.D., B.I. Koura, A. Vastolo, M.F. Chiacchio, P. Vitaglione, L.H. Dossa, M.I. Cutrignelli, and S. Calabrò. 2024. Sustainable ruminant nutrition in West Africa by *in vitro* characterization of cashew apple by-products. Heliyon 10(18):e37737. doi: https://doi.org/10.1016/j.heliyon.2024.e37737 39315231 PMC11417178

[CIT0022] Koura, B.I., A. Vastolo, D.D. Kiatti, M.I. Cutrignelli, M. Houinato, and S. Calabrò. 2022. Nutritional value of climate-resilient forage species sustaining peri-urban dairy cow production in the coastal grasslands of Benin (West Africa). Animals 12(24):3550. doi: https://doi.org/10.3390/ani12243550 36552468 PMC9774299

[CIT0023] Koura, B.I., F.P. Yassegoungbe, and L.H. Dossa. 2024. Production systems and strategies of peri-urban goat and sheep farmers for dry season feeding: a case study from Benin (West Africa). Cogent Food Agric. 10(1):2356934. doi: https://doi.org/10.1080/23311932.2024.2356934

[CIT0044] Mapiye, O., O.C. Chikwanha, G. Makombe, and K. Dzama. 2020. Livelihood, Food and Nutrition Security in Southern Africa: What Role Do Indigenous Cattle Genetic Resources Play? Diversity 12(2):74. doi: https://doi.org/10.3390/d12020074

[CIT0024] Mawdsley, E. 2019. South–South cooperation 3.0? Managing the consequences of success in the decade ahead. Oxford Devel. Stud. 47(3):259–274. doi: https://doi.org/10.1080/13600818.2019.1585792

[CIT0025] Nchanji, E.B., K. Kamunye, and C. Ageyo. 2023. Thematic evidencing of youth-empowering interventions in livestock production systems in Sub-Sahara Africa: a systematic review. Front. Sustain. Food Syst. 7:1176652. doi: https://doi.org/10.3389/fsufs.2023.1176652

[CIT0026] Nicol, C.J. 2021. A grand challenge for animal science: multiple goals—convergent and divergent. Front. Anim. Sci. 2(2):640503. doi: https://doi.org/10.3389/fanim.2021.640503

[CIT0027] OIE (World Organisation for Animal Health). 2020a. Global strategies for animal disease control– [accessed April 24, 2025]. https://www.woah.org/app/uploads/2021/03/bull-2013-3-eng.pdf

[CIT0028] OIE (World Organisation for Animal Health). 2020b. Animal health status report– [accessed April 24, 2025]. https://www.report2020oie.fr/en/

[CIT0029] Oloo, R.D., J.M. Ojango, C.C. Ekine-Dzivenu, G. Gebreyohanes, R. Mrode, O.A. Mwai, and M.G. Chagunda. 2023. Enhancing individual animal resilience to environmental disturbances to address low productivity in dairy cattle performing in sub-Saharan Africa. Front. Anim. Sci. 4:1254877. https://doi.org/10.3389/fanim.2023.1254877

[CIT0030] Ouédraogo, D., A. Soudré, B. Yougbaré, S. Ouédraogo-Koné, B. Zoma-Traoré, N. Khayatzadeh, and J. Sölkner. 2021. Genetic improvement of local cattle breeds in West Africa: a review of breeding programs. Sustainability 13(4):2125. https://doi.org/10.3390/su13042125

[CIT0031] Rahman, M.T., M. Saiful Islam, A.A. Shehata, B. Shereen, M.H. Hafez, E.I. Azhar, A.F. Khafaga, and F. Bovera. 2022. Influence of COVID-19 on the sustainability of livestock performance and welfare on a global scale. Trop. Anim. Health Prod. 54:309. doi: https://doi.org/10.1007/s11250-022-03256-x PMC9483476 36114917

[CIT0032] Rekaya, R., and S.E. Aggrey. 2020. Landscape genomics: application in poultry breeding. In: Aggrey, S.E., H. Zhou, M. Tixier-Boichard, and D. Rhoads, editors. Advances in poultry genetics and genomics. London, England: Burleigh Dodds Science Publishing. ISBN 9781786763242.

[CIT0033] SADC. 2019. Regional agricultural policy– [accessed April 28, 2025]. https://www.nepad.org/publication/sadc-regional-agricultural-policy-0

[CIT0034] SASAS. 2021. About animal science– [accessed April 24, 2005]. https://www.sasas.co.za/about-animal-sciences/

[CIT0041] Sharaf, A., L.T. Nesengani, I. Hayah, J.O. Kuja, S. Mdyogolo, T.C. Omotoriogun, B.A. Odogwu, G. Beedessee, R.M. Smith, A. Barakat, et al 2024. Establishing African genomics and bioinformatics programs through annual regional workshops. Nat. Genet. 56(8):1556–1565. doi: https://doi.org/10.1038/s41588-024-01807-6 38977855

[CIT0036] Shehata, A.A., Y.A. Attia, M.T. Rahman, S. Basiouni, H.R. El-Seedi, E.I. Azhar, A.F. Khafaga, and H.M. Hafez. 2022. Diversity of coronaviruses with particular attention to the interspecies transmission of SARS-CoV-2. Animals 12(3):378. doi: https://doi.org/10.3390/ani12030378 35158701 PMC8833600

[CIT0037] Van der Poel, A.F.B., M.R. Abdollahi, H. Cheng, R. Colovic, L.A. Den Hartog, D. Miladinovic, and W.H. Hendriks. 2020. Future directions of animal feed technology research to meet the challenges of a changing world. Anim. Feed Sci. Technol. 270:114692. doi: https://doi.org/10.1016/j.anifeedsci.2020.114692

[CIT0038] Vilas-Boas, J., L. Klerkx, and R. Lie. 2022. Connecting science, policy, and practice in agri-food system transformation: the role of boundary infrastructures in the evolution of Brazilian pig production. J. Rural Stud. 89:171–185. doi: https://doi.org/10.1016/j.jrurstud.2021.11.025

[CIT0039] Vlaicu, P.A., M.A. Gras, A.E. Untea, N.A. Lefter, and M.C. Rotar. 2024. Advancing livestock technology: intelligent systemization for enhanced productivity, welfare, and sustainability. Agric. Eng. 6(2):1479–1496. doi: https://doi.org/10.3390/agriengineering6020084

[CIT0040] Wong, M. 2023. Seven ways to sustainably transform livestock systems in Africa– [accsessed April 24, 2025]. https://whylivestockmatter.org/articles/seven-ways-sustainably-transform-livestock-systems-africa

